# Internal Tandem Duplication in FLT3 Attenuates Proliferation and Regulates Resistance to the FLT3 Inhibitor AC220 by Modulating p21^Cdkn1a^ and Pbx1 in Hematopoietic Cells

**DOI:** 10.1371/journal.pone.0158290

**Published:** 2016-07-07

**Authors:** Mariko Abe, Louis M. Pelus, Pratibha Singh, Tomohiro Hirade, Chie Onishi, Jamiyan Purevsuren, Takeshi Taketani, Seiji Yamaguchi, Seiji Fukuda

**Affiliations:** 1 Department of Pediatrics, Shimane University School of Medicine, Izumo, Shimane, Japan; 2 Department of Microbiology/Immunology, Indiana University School of Medicine, Indianapolis, Indiana, United States of America; 3 Department of Oncology/Hematology, Shimane University School of Medicine, Izumo, Shimane, Japan; 4 Division of Blood Transfusion, Shimane University Hospital, Izumo, Shimane, Japan; B.C. Cancer Agency, CANADA

## Abstract

Internal tandem duplication (ITD) mutations in the *Fms-related tyrosine kinase 3 (FLT3)* gene (*FLT3*-ITD) are associated with poor prognosis in patients with acute myeloid leukemia (AML). Due to the development of drug resistance, few FLT3-ITD inhibitors are effective against FLT3-ITD^+^ AML. In this study, we show that FLT3-ITD activates a novel pathway involving p21^Cdkn1a^ (p21) and pre-B cell leukemia transcription factor 1 (Pbx1) that attenuates FLT3-ITD cell proliferation and is involved in the development of drug resistance. FLT3-ITD up-regulated p21 expression in both mouse bone marrow c-kit^+^-Sca-1^+^-Lin^-^ (KSL) cells and Ba/F3 cells. The loss of p21 expression enhanced growth factor-independent proliferation and sensitivity to cytarabine as a consequence of concomitantly enriching the S+G_2_/M phase population and significantly increasing the expression of Pbx1, but not Evi-1, in FLT3-ITD^+^ cells. This enhanced cell proliferation following the loss of p21 was partially abrogated when Pbx1 expression was silenced in FLT3-ITD^+^ primary bone marrow colony-forming cells and Ba/F3 cells. When FLT3-ITD was antagonized with AC220, a selective inhibitor of FLT3-ITD, *p21* expression was decreased coincident with *Pbx1* mRNA up-regulation and a rapid decline in the number of viable FLT3-ITD^+^ Ba/F3 cells; however, the cells eventually became refractory to AC220. Overexpressing p21 in FLT3-ITD^+^ Ba/F3 cells delayed the emergence of cells that were refractory to AC220, whereas p21 silencing accelerated their development. These data indicate that FLT3-ITD is capable of inhibiting FLT3-ITD^+^ cell proliferation through the p21/Pbx1 axis and that treatments that antagonize FLT3-ITD contribute to the subsequent development of cells that are refractory to a FLT3-ITD inhibitor by disrupting p21 expression.

## Introduction

The cyclin-dependent kinase inhibitor p21^Cdkn1a^ (p21) was originally identified as a mediator of p53-induced growth arrest [1,2] and regulates the proliferation of diverse cell types by modulating cell cycle, apoptosis and differentiation [3–5]. However, cells in which p53 is not activated still utilize p21-dependent cell cycle control [6–8]. P21 is highly expressed in hematopoietic stem cells (HSCs) and maintains quiescence [8]. The loss of p21 in HSCs increases cell cycle progression but has only a marginal effect on marrow cellularity or peripheral blood cell counts [8]. In contrast, p21 can facilitate the proliferation of normal hematopoietic progenitor cells (HPCs) ex vivo following stimulation with hematopoietic growth factors [9–12]. These findings suggest that p21 has a differentiation stage-specific function in the hematopoietic system. In addition to regulating the cell cycle [1,2,6,7,13], p21 can modulate the activity of a number of transcription factors and co-activators, such as Stat3 [13,14], estrogen receptorα[15], (Ccaat-enhancer-binding protein α (C/EBPα) [16,17] and c-Myc [18], suggesting that it may regulate cell fate by influencing gene expression [19].

Internal tandem duplication (ITD) mutations in the *Fms-related tyrosine kinase 3 (FLT3)* gene (*FLT3*-ITD) are found in 20–30% of patients with acute myeloid leukemia (AML) and are associated with a poor prognosis [20,21]. Few FLT3-ITD inhibitors are effective against FLT3-ITD^+^ AML due to the emergence of drug-resistant cells [22,23]. For instance, AC220 (Quizartinib), a second-generation class III tyrosine kinase inhibitor (TKI), which has been used to treat FLT3-ITD^+^ AML in clinical trials, is a more potent and specific FLT3-ITD inhibitor than other TKIs [22,24]; however, prolonged exposure to AC220 could generate FLT3-ITD^+^ cells that are resistant to AC220 [25]. The mechanisms responsible for drug resistance include the acquisition of mutations in the *FLT3* gene, the activation of other pro-survival pathways and microenvironment-mediated resistance [22,23]; however, additional mechanisms responsible for the drug resistance of FLT3-ITD^+^ AML cells remain to be investigated. Previous studies have shown that FLT3-ITD enhances p21 expression through Stat5 [26], whereas the FLT3-ITD inhibitor SU5614 decreases p21 expression in Ba/F3 cells expressing FLT3-ITD [27]. P21 down-regulation by the FLT3-ITD inhibitor suggests that treatments that antagonize FLT3-ITD may destroy p21 function and aberrantly affect FLT3-ITD^+^ cell proliferation. However, the functional role of p21 in FLT3-ITD signaling and FLT3-ITD-induced drug resistance remains unknown.

In the present study, we identified a p21 signaling pathway downstream of FLT3-ITD that negatively affects proliferation and is associated with drug resistance in FLT3-ITD^+^ cells. An analysis of the genes that are modulated by p21 deletion in FLT3-ITD-transformed HPCs revealed that p21 modulates the expression of pre-B cell leukemia transcription factor 1 (Pbx1), a proto-oncogene that critically regulates HSC and HPC function [28,29]. Silencing Pbx1 and p21 expression in FLT3-ITD-transformed HPCs revealed that the interaction between FLT3-ITD-activated p21 and Pbx1 negatively regulated FLT3-ITD^+^ HPC proliferation. In addition, the down-regulation of p21 resulting from FLT3-ITD inhibition by AC220 accelerated the emergence of FLT3-ITD^+^ cells that were resistant to AC220. This study is the first report to show how treatments targeting FLT3-ITD can lead to drug resistance.

## Materials and Methods

### Animals

Specific pathogen-free female C57BL/6 mice, 6–8 weeks of age, were purchased from CLEA Japan, Inc. (Tokyo, Japan). P21^-/-^ mice were kindly provided by Dr. H.E. Broxmeyer of the Indiana University School of Medicine [9,10]. Survivin^fx/fx^ mice and Tx-Cre Survivin^fx/fx^ mice have been described previously [30]. The IACUC of the Shimane University School of Medicine (Permit Numbers IZ21-24, IZ21-25, and IZ21-26) and the Indiana University School of Medicine (Study Number 2939) approved all of the experimental procedures.

### Antibodies and cytokines

Anti-FcγIII/II receptor antibody, allophycocyanin (APC)-conjugated anti-mouse c-kit antibody (clone 2B8), phycoerythrin (PE)-conjugated Annexin V and PE-Cy7-conjugated anti-Sca-1 antibody (E13-161.7), along with streptavidin-APC-Cy7, rat IgG2a, rat IgG2b, 7-actinomycin-D (7-AAD) and anti-p27 monoclonal antibodies were all purchased from BD Biosciences (San Diego, CA). Biotinylated antibodies against lineage markers, including CD5, B220, CD11b, Gr-1, 7–4 and Ter119, were purchased from Miltenyi Biotec (Auburn, CA). The anti-phospho-FLT3 antibody (Tyr591 33G6) and PE-conjugated anti-rabbit IgG (Fab’) were obtained from Cell Signaling (Danvers, MA). Hoechst 33342 was purchased from Molecular Probes (Eugene, OR). Recombinant murine (rm) interleukin-3 (IL-3), recombinant human (rh) Fms-related tyrosine kinase 3 (FLT3) ligand (FL) and rh-thrombopoietin (TPO) were obtained from R&D Systems (Minneapolis, MN). Rm-stem cell factor (SCF) was purchased from BioVision Research Products (Mountain View, CA). Pyronin Y, AG1296, PD98059 and LY294002 were obtained from Wako Pure Chemical Industries (Osaka, Japan). AC220 (Quizartinib) was obtained from Selleckchem.com (Houston, TX). An Akt inhibitor, etoposide, and pifithrin-α were purchased from Calbiochem (Gibbstown, NJ). H89 was purchased from Upstate Biotechnology (Lake Placid, NY). The anti-actin (I-19) and anti-p21 (F-5) antibodies were obtained from Santa Cruz Biotechnology (Santa Cruz, CA).

### Cell culture, plasmid transfection, retroviral transduction and shRNA knockdown

Ba/F3 cells expressing wild-type human FLT3 or FLT3-ITD have been described previously [30–32]. Retroviral transduction of human wild-type *FLT3* and N51-*FLT3*-ITD in an MSCV-IRES-EGFP vector into mouse bone marrow cells was performed as previously described [9,30]. After sequential infections, the GFP^+^ cells were sorted using a FACSAria II flow cytometer (BD Biosciences), and 25,000 GFP^+^ cells were cultured in semisolid methylcellulose or 0.3% agar with 30% heat-inactivated fetal bovine serum (HI-FBS) without hematopoietic growth factors. Colony formation was scored after 7 or 14 days. In replicate liquid cultures, cells were stained for c-kit, Sca-1 and standard lineage markers at the time of plating or after incubation in liquid culture. For shRNA-mediated knockdown of *Pbx1*, bone marrow cells from the p21^+/+^ and p21^-/-^ mice were transduced with a control shRNA or *Pbx1* shRNA (shRNA-1 or shRNA-2) using a pSINsi-mU6 plasmid (TAKARA Biotechnology, Otsu, Japan). The sequences of *Pbx1* shRNA-1 and shRNA-2 were 5’-CGA TCA ATG CAT ATT TGC A-3’ and 5’-GGA GCA TTC CGA CTA CAG A-3’, respectively. To transduce shRNAs targeted against *p21* and/or *Pbx1* into the Flt3-ITD^+^ Ba/F3 cells, non-transfected Ba/F3 cells were transduced with MSCV-*FLT3*-ITD (N51)-EGFP using a retrovirus. The GFP-positive cells were sorted and electroporated with a *p21*-specific shRNA that had been cloned into the pBAsi-mU6 Pur DNA vector (TAKARA Biotechnology). The sequence of the *p21* shRNA was 5’-GCA GAT TGG TCT GCA A-3’. Stable transformants were selected with 3 μg/ml puromycin and frozen for storage. The cells were subsequently transfected with a pSINsi-mU6 plasmid containing a *Pbx1* shRNA, and G418-resistant cells were selected and used for the analyses.

### cDNA microarrays and quantitative RT-PCR

Lineage-depleted bone marrow cells obtained from the p21^+/+^ and p21^-/-^ mice were transduced with MSCV-IRES-EGFP containing N51-*FLT3*-ITD3. Following transduction, the GFP^+^, c-kit^+^, Sca-1^+^, lineage-negative (KSL) cells were isolated by fluorescence-activated cell sorting (FACS). Freshly isolated control KSL cells from the same donors were used for the subsequent comparisons. The sorted cells were subjected to a differential mRNA microarray analysis, which was performed by Miltenyi Biotec (Auburn, CA). Briefly, 250 ng of each cDNA was used as a template for Cy3 and Cy5 labeling. Equal amounts of the corresponding Cy3- and Cy5-labeled cDNAs from the p21^+/+^ and p21^-/-^ cells were combined and hybridized overnight (17 h at 65°C) to Agilent Whole Mouse Genome Oligo Microarrays according to the manufacturer’s protocol. The microarray data have been deposited in the Gene Expression Omnibus (www.ncbi.nlm.nih.gov/geo/: GSE75200). In separate experiments, the total RNAs isolated from sorted p21^+/+^ and p21^-/-^ KSL cells, with or without FLT3-ITD, were reverse transcribed and mixed with Power SYBR Green PCR SuperMix (Applied Biosystems, Foster City, CA) for quantitative RT-PCR (Q-RT-PCR). The PCR primers were: *Pbx1*, 5’-AAGCCGGACCAGGCCCATCT-3’ and 5’-GGCCTCGCACGTGCTCTGTT-3’; *Evi-1*, 5’-GCAGCGACATGTGCGCAACA-3’ and 5’-GAGGCGAGGACGTTGCCGTC-3’; *p21*, 5’-GTACTTCCTCTGCCCTGC-3’ and 5’-AGAGTGCAAGACAGCGACAA-3’; and *Hprt*, 5’-TGGACAGGACTGAAAGACTTGCTCG-3’ and 5’-GGCCACAATGTGATGGCCTCCC-3’. The PCR cycling parameters were 95°C for 10 min, followed by 50 cycles at 95°C for 15 s and 60°C for 1 min. The differences in the mRNA levels were calculated using the ΔCT method and were normalized to *Hprt*.

### Statistical analysis

The data are expressed as the means ± standard errors of the mean (SEM). Significant differences were determined using a two-tailed Student’s t-test in Microsoft Excel^™^ (Microsoft Corp., Seattle, WA).

## Results

### FLT3 signaling up-regulates p21^Cdkn1a^ expression in Ba/F3 cells through the p53, PI3 kinase and PKA pathways

**Fig 1:**
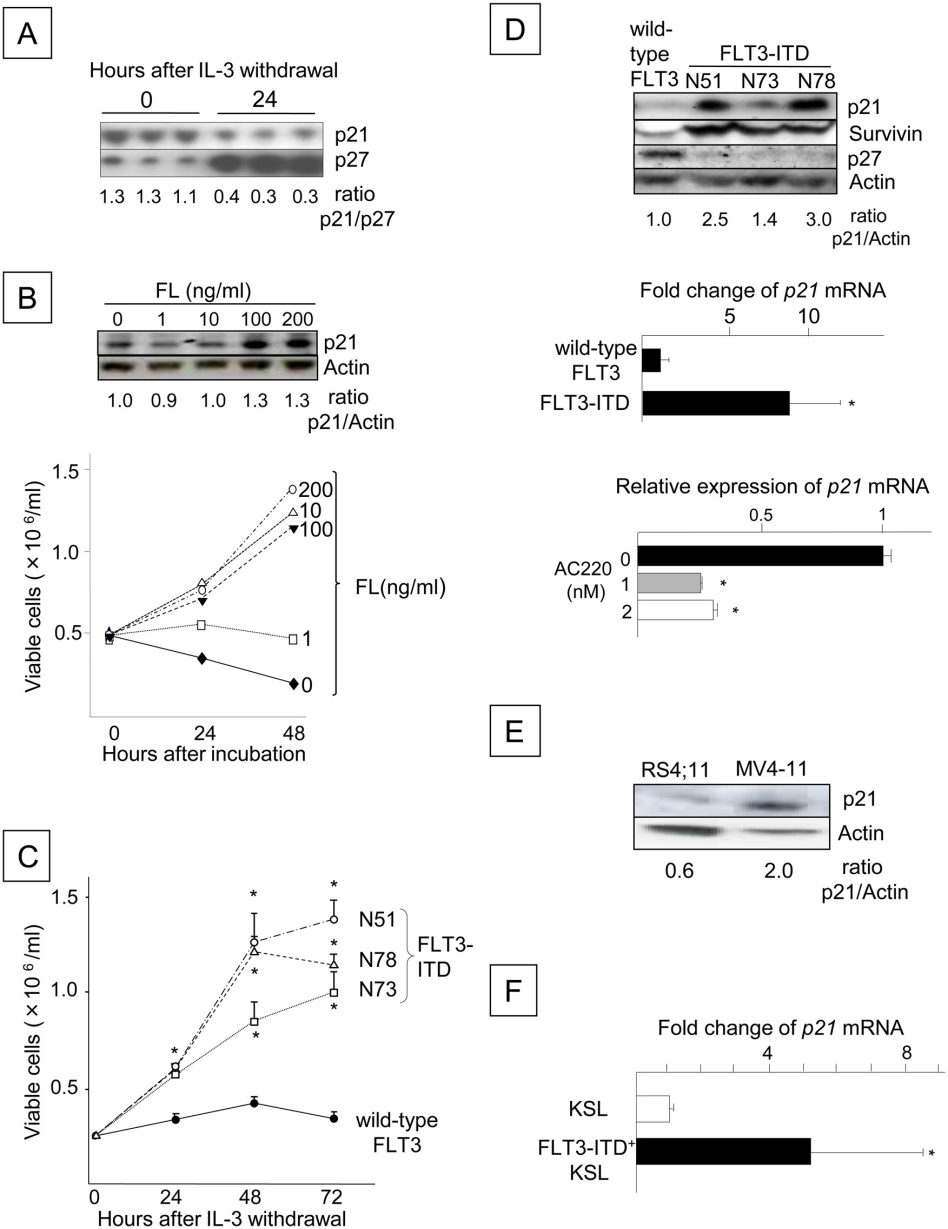
FLT3 Signaling Up-regulates p21^Cdkn1a^ Expression in Ba/F3 Cells. (A) Expression of p21 and p27 in Ba/F3-FLT3 cells before and after IL-3 withdrawal. The blots are from triplicate cultures. The total number of viable cells was enumerated at the time of harvest using the trypan blue exclusion assay, and lysates from 5x10^5^ viable cells were loaded into each lane. The p21/p27 ratio was determined by densitometry and is shown beneath the blot. (B) The number of viable Ba/F3-FLT3 cells stimulated with or without FL. Following IL-3 withdrawal for 16 h, the cells were resuspended in 10% FBS/RPMI-1640 at a concentration of 5x10^5^ cells/ml with FL concentrations ranging from 0 to 200 ng/ml. The number of viable cells was counted 24 and 48 h after incubation. P21 protein expression was analyzed by Western blotting at 48 h (top). The data are representative of two experiments with identical results. (C) Proliferation of Ba/F3 cells expressing wild-type or FLT3-ITD. The cells were washed three times and cultured for 72 h in 1% FBS/RPMI-1640, and the numbers of viable Ba/F3 cells expressing wild-type or FLT3-ITD derived from 3 different patients (N51, N73 and N78) were counted every 24 h (*: P < 0.05). The data shown are from one of three experiments that were performed in triplicate with identical results. (D) The upper panel shows the expression of the p21, Survivin and p27 proteins, as analyzed by Western blotting. The level of p21 relative to actin is shown beneath the blot. The middle panel shows the relative *p21* mRNA expression levels in Ba/F3 cells expressing wild-type or N51-FLT3-ITD, which were determined using quantitative RT-PCR. The presented data are the averages of two independent experiments (*: P < 0.05). The lower panel shows the *p21* mRNA expression levels in FLT3-ITD (N51) Ba/F3 cells incubated with 1 or 2 nM AC220 for 48 h compared to the control cells, which were incubated with dimethyl sulfoxide (DMSO) alone (N = 3, *: P < 0.05). (E) The blot shows p21 protein expression in RS4;11 cells and MV4-11 cells, as determined by Western blot analysis. (F) The *p21* mRNA expression levels in FLT3-ITD^+^ KSL cells compared to freshly isolated bone marrow KSL cells. C57BL/6 mouse bone marrow cells were retrovirally transduced with N51-*FLT3*-ITD using the MSCV-IRES-EGFP vector and were cultured for 2 weeks without hematopoietic growth factors. The GFP^+^ KSL cells were sorted by FACS, and *p21* mRNA expression was compared with freshly isolated KSL cells from the same donor. The presented data are the averages of three independent experiments (*: P < 0.005).

We previously showed that *FLT3*-ITD mutations increase Survivin expression in mouse Ba/F3 cells [30] and that Survivin enhances the proliferation of primary mouse HPCs through p21-dependent and p21-independent mechanisms [9]. Although p21 negatively regulates cell proliferation in a variety of cell systems [6–8], these findings suggest that p21 may positively regulate FLT3-ITD signaling, similar to Survivin. We first investigated the association between p21 expression and cell proliferation in response to normal FLT3 and FLT3-ITD signaling. In IL-3-dependent Ba/F3 cells expressing wild-type human FLT3 (Ba/F3-FLT3), IL-3 withdrawal, which generally induces G_1_ arrest, resulted in down-regulation of the p21 ^Cdkn1a^ protein and up-regulation of p27 ^Cdkn1b^, a protein that normally inhibits the G_1_/S transition [33,34] (Fig 1A). The stimulation of Ba/F3-FLT3 cells with FLT3 ligand (FL) concomitantly increased p21 protein levels and total cell number (Fig 1B). The ectopic expression of FLT3-ITD in Ba/F3 cells (FLT3-ITD^+^ Ba/F3) significantly enhanced cell proliferation (Fig 1C). These changes were coincident with the up-regulation of p21 and Survivin levels and a reduction in p27 ^Cdkn1b^ levels compared to cells expressing wild-type FLT3 (Fig 1D, upper panel). Consistent with the up-regulation of the p21 protein, *p21* mRNA expression was significantly increased in FLT3-ITD^+^ Ba/F3 cells compared to cells expressing wild-type FLT3 (Fig 1D, middle panel). However, the selective FLT3-ITD inhibitor AC220 [24] significantly decreased *p21* mRNA expression in FLT3-ITD^+^ Ba/F3 cells (Fig 1D, lower panel). Similarly, treatment of FLT3-ITD^+^ cells with AG1296, which inhibits FLT3 kinase [35], decreased p21 expression (not shown). Consistent with the FLT3-ITD^+^ Ba/F3 cells, increased levels of the p21 protein were also observed in FLT3-ITD^+^ MV4-11 human acute leukemia cells compared to RS4;11 cells expressing wild-type FLT3 (Fig 1E). The transduction of *FLT3*-ITD into mouse bone marrow cells and culture in the absence of hematopoietic growth factors for 14 days resulted in an expansion of c-kit^+^, Sca-1^+^, lineage-negative (KSL) cells as previously reported [30] (not shown), along with a significant up-regulation of the *p21* mRNA in KSL cells (Fig 1F). These data indicate that p21 expression is up-regulated by FLT3 signaling, and similar to Survivin, the increase is associated with enhanced or accelerated cell proliferation.

**Fig 2:**
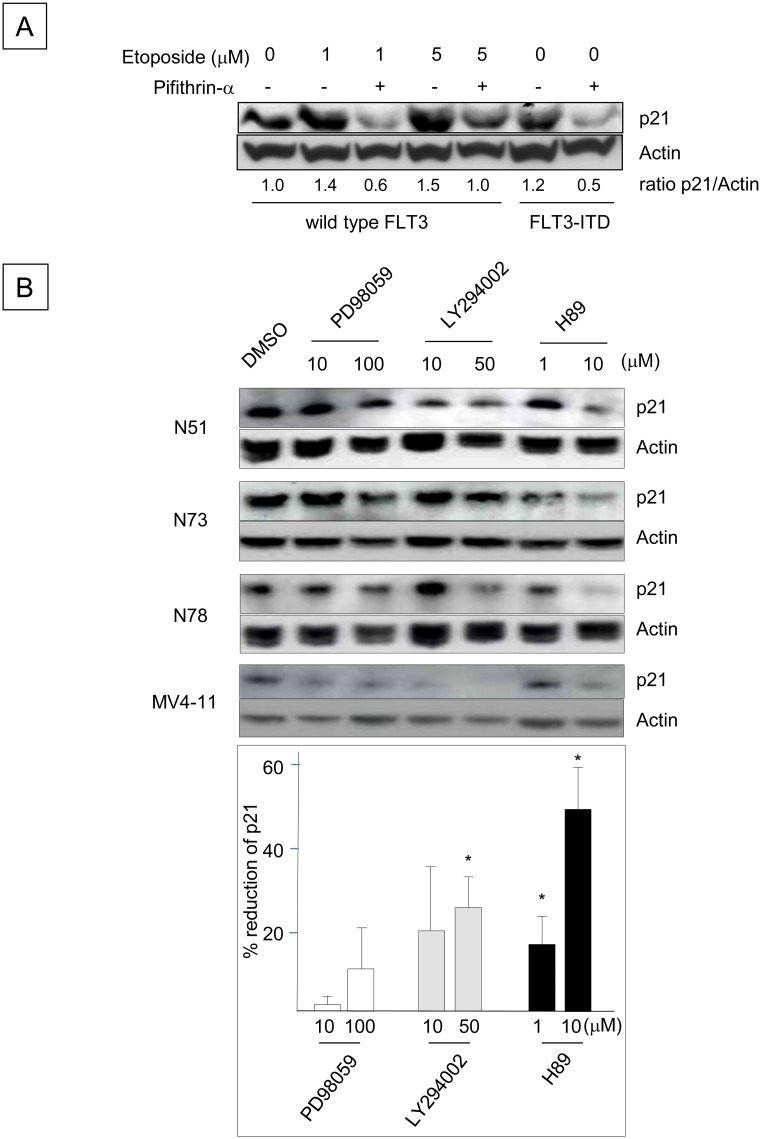
Up-regulation of p21 Expression Induced by FLT3-ITD is Mediated through the p53, PI3 Kinase and PKA Pathways. (A) Ba/F3 cells expressing wild-type FLT3 were incubated with 20 μM pifithrin-α for 24 h, followed by exposure to 1 or 5 μM etoposide for 2.5 h. Ba/F3 cells expressing N51-FLT3-ITD were also incubated with pifithrin-α for 24 h. The cell lysates were subjected to Western blot analysis to determine the p21 protein expression levels. (B) Ba/F3 cells containing N51, N73 and N78 FLT3-ITD were incubated with the indicated concentrations of DMSO, PD98059, LY294002 or H89 (all of which were diluted in DMSO) in 10% FBS/RPMI-1640 supplemented with 0.1 ng/ml rmIL-3 for 24 h. Human MV4-11 leukemia cells harboring endogenous *FLT3*-ITD were treated in the same manner and were incubated in 10% FBS/RPMI-1640. The p21 expression levels (relative to actin) were quantitated by Western blot analysis and densitometry. The histogram beneath the blot indicates the percentages by which p21 expression was reduced by the inhibitors, which were averaged in 4 wells and determined by a comparison with the DMSO control (*: P < 0.05).

Because p21 is one of the major transcriptional targets of p53 [1,36], we next investigated whether p53 is involved in the up-regulation of p21 induced by FLT3-ITD (Fig 2A). Incubation of BaF3 cells with etoposide, which activates p53 [37], increased p21 protein levels, indicating that p53 is functional in Ba/F3 cells. Etoposide-enhanced p21 expression was abrogated by the p53 inhibitor pifithrin-α [1,38], and FLT3-ITD^+^ Ba/F3 cells treated with pifithrin-α also down-regulated p21 expression in the absence of etoposide. These results suggest that the up-regulation of p21 in FLT3-ITD^+^ Ba/F3 cells is mediated by p53. To identify additional signaling pathways that regulate the p21 expression induced by FLT3-ITD, Ba/F3 cells expressing several different human FLT3-ITD constructs were incubated with inhibitors of mitogen-activated protein kinase (MAPK)^p42/p44^ (PD98059), phosophoinositide-3 (PI3) kinase (LY294002) and protein kinase A (PKA) (H89). Although the MAPK inhibitor had little effect on p21 protein expression, the PI3 kinase and PKA inhibitors reduced p21 levels. Consistent with the results obtained using FLT3-ITD^+^ Ba/F3 cells, the PI3 kinase and PKA inhibitors reduced p21 expression in human FLT3-ITD^+^ MV4-11 leukemia cells (Fig 2B). These data indicate that the p53, PI3 kinase and PKA pathways mediate the FLT3-ITD-induced up-regulation of p21.

### FLT3-ITD attenuates the growth factor-independent proliferation and cell cycle progression of hematopoietic progenitor cells by up-regulating p21^Cdkn1a^ expression

**Fig 3:**
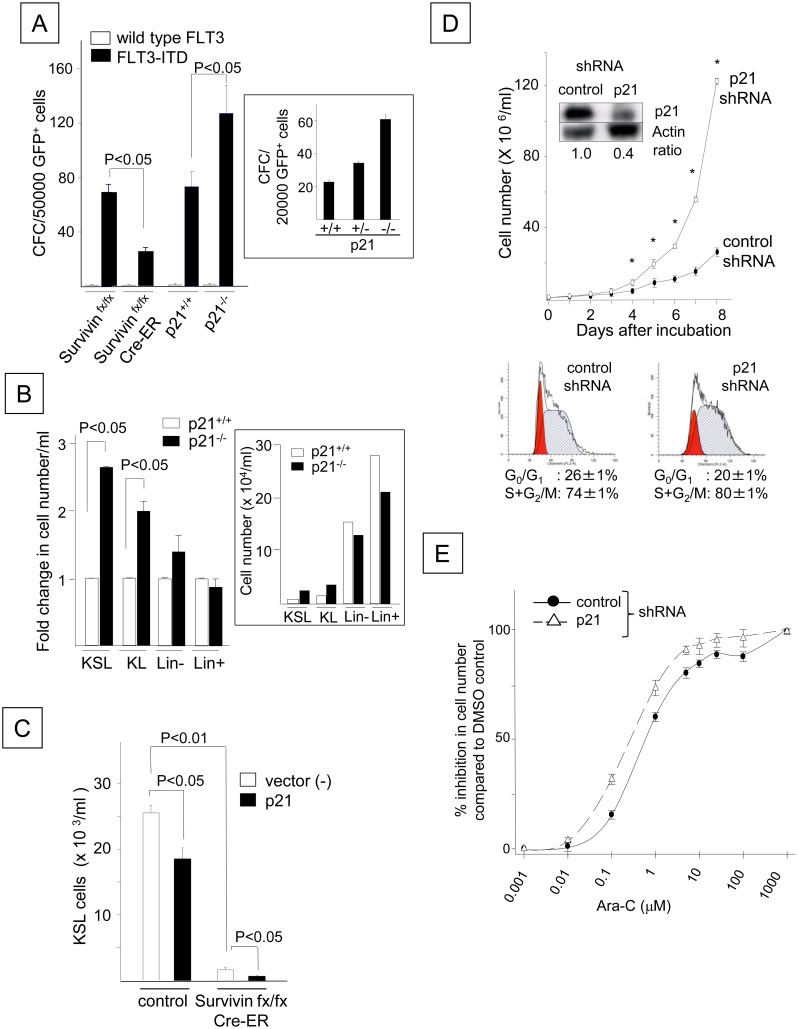
FLT3-ITD Attenuates the Growth Factor-independent Proliferation and Cell Cycle Progression of Hematopoietic Progenitor Cells by Up-regulating p21^Cdkn1a^ Expression. (A) Growth factor-independent proliferation of CFCs in mouse bone marrow cells lacking p21 or Survivin and transduced with *FLT3*-ITD. Bone marrow cells from Survivin^fx/fx^, Cre-ER Survivin^fx/fx^, p21^+/+^ or p21^-/-^ mice were retrovirally transduced with wild-type *FLT3* or N51-*FLT3*-ITD. A total of 500,000 GFP^+^ cells were plated on either 0.3% agar or methylcellulose medium containing 30% FBS in the absence of hematopoietic growth factors. To delete the *Survivin* gene, cells from Cre-ER Survivin^fx/fx^ or Survivin^fx/fx^ mice were cultured in the presence of 1 μM 4-hydroxy tamoxifen at the time of plating. The colonies were enumerated after 14 days. The presented data are the averages of 9 experiments for p21 deletion and 2 experiments for Survivin deletion (P < 0.05). The numbers of CFCs generated by p21^+/-^ cells transduced with *FLT3*-ITD compared to those generated by p21^+/+^ and p21^-/-^ cells are shown in the lower panel. The data shown were obtained from one of two experiments that yielded identical results. (B) The proliferation of bone marrow c-kit^+^, Sca-1^+^, lineage^-^ (KSL), c-kit^+^, lineage^-^ (KL), lineage^-^ (Lin^-^) or lineage^+^ (Lin^+^) cells transduced with *FLT3*-ITD derived from p21^+/+^ and p21^-/-^ mice. Following transduction, the GFP^+^ cells were sorted and cultured in 10% FBS/IMDM in the absence of hematopoietic growth factors. The cells were enumerated 7 days after culture. The fold change in the number of FLT3-ITD^+^ p21^-/-^ cells compared to the FLT3-ITD^+^ p21^+/+^ cells is shown. The presented data are the averages of three experiments (P < 0.05). The cell number from one representative experiment is shown in the lower panel. (C) The effect of ectopic p21 expression on FLT3-ITD^+^ KSL cell proliferation. Bone marrow cells from control Survivin^fx/fx^ mice or Cre-ER Survivin^fx/fx^ mice were transduced with N51-*FLT3*-ITD and *p21* using a MIEG3 vector or a negative MIEG3 vector control. Following transduction, the GFP^+^ cells were cultured for 2 weeks in 10% FBS/IMDM containing 1μM 4-hydroxy tamoxifen at a density of 5 x 10^5^ cells/ml. The cells were stained for c-kit, Sca-1 and lineage markers to quantify the number of KSL cells. The data shown were obtained from one of two experiments that were analyzed in triplicate. (D) Ba/F3 cells expressing FLT3-ITD (N51) were transduced with either a *p21* shRNA or a control shRNA and were cultured in 1% FBS/RPMI without any growth factors. The viable cells were enumerated using the trypan blue exclusion assay. The data shown were obtained from one of two experiments that were performed in quadruplicate with identical results (*: P < 0.01). The expression levels of the p21 protein in both cell lines are shown in the inset. The lower panel shows a cell cycle histogram of FLT3-ITD^+^ Ba/F3 cells transduced with a control shRNA (left plot) and the p21 shRNA (right plot). The histogram represents one of three experiments with identical results. (E) Dose-dependent growth inhibition by Ara-C in N51-FLT3-ITD-Ba/F3 cells transduced with the *p21* shRNA and control cells. The cells were cultured in the presence of DMSO alone (control) or increasing doses of Ara-C in 1% FBS/RPMI-1640 without any growth factors for 24 hours. The y-axis represents the % inhibition of viable cells incubated with Ara-C compared to DMSO. The data shown represent one of three experiments with identical results.

The concurrent enhancement of cell proliferation and p21 up-regulation by FLT3-ITD suggested that p21 may positively regulate FLT3-ITD-potentiated cell proliferation, which was similar to our previous observations for the endogenous anti-apoptotic protein Survivin [30]. Therefore, we compared the functional roles of p21 and Survivin in the FLT3-ITD-induced growth factor-independent proliferation of HPCs. Although wild-type FLT3 failed to support the proliferation of colony-forming cells (CFCs), FLT3-ITD overexpression in control Survivin^fx/fx^ or p21^+/+^ bone marrow cells resulted in CFC proliferation in the absence of hematopoietic growth factors (Fig 3A). Conditional deletion of Survivin reduced the growth factor-independent proliferation of *FLT3*-ITD-transduced CFCs, as previously reported (Cre-ER Survivin^fx/fx^ in Fig 3A) [30]. In contrast, deletion of the *p21* gene increased the proliferation of CFCs transduced with *FLT3*-ITD (Fig 3A). An intermediate number of CFCs was generated by p21^+/-^ cells transduced with *FLT3*-ITD, indicating that the effect of p21 was gene dosage-dependent (Fig 3A, inset). Similar to CFCs, the growth factor-independent proliferation of FLT3-ITD^+^ KSL and c-kit^+^, lineage-negative (KL) cells was further enhanced by the deletion of p21 (P < 0.05, Fig 3B). In contrast, the loss of p21 did not significantly affect cell number or the proportion of lineage-positive and lineage-negative cells. Although no difference was observed in the number of apoptotic cells between the FLT3-ITD^+^ p21^+/+^ KL and FLT3-ITD^+^ p21^-/-^ KL cells, the enhancement of FLT3-ITD^+^ cell proliferation by p21 deletion was associated with a marginal but significant increase in the number of cells in S+G_2_/M phase (18.2 ± 0.5%) compared to p21^+/+^ cells (15.3 ± 0.8%, P < 0.05, N = 3), concomitant with increased levels of Ki-67, a marker for cycling cells (1.5 ± 0.1-fold increase, P < 0.01, N = 3). In contrast, ectopic p21 overexpression decreased the number of growth factor-independent FLT3-ITD^+^ KSL cells derived from the control cells compared to cells expressing FLT3-ITD alone (P < 0.05). The loss of Survivin (Cre-ER Survivin^fx/fx^) further reduced the number of FLT3-ITD^+^ KSL cells overexpressing p21 (Fig 3C). Similarly, treatment with a *p21*-specific shRNA increased the growth factor-independent proliferation of FLT3-ITD^+^ Ba/F3 cells compared to treatment with a control shRNA and was coincident with a marginal but significant increase in the number of cells in S+G_2_/M phase (Fig 3D). Moreover, p21 silencing increased the sensitivity of FLT3-ITD^+^ Ba/F3 cells to the cell cycle-specific chemotherapeutic agent cytarabine (Ara-C) (Fig 3E) [39]. The 50% inhibitory concentrations (IC50s) of Ara-C were 0.6 and 0.27 μM in the control FLT3-ITD^+^ Ba/F3 cells and those transduced with p21 shRNA, respectively. These data suggest that the FLT3-ITD-mediated up-regulation of p21 inhibits FLT3-ITD^+^ cell proliferation and confers resistance to chemotherapy by delaying cell cycle progression.

### Blocking p21 by FLT3-ITD inhibition facilitates the emergence of FLT3-ITD^+^ cells that are refractory to the FLT3-ITD inhibitor AC220

**Fig 4:**
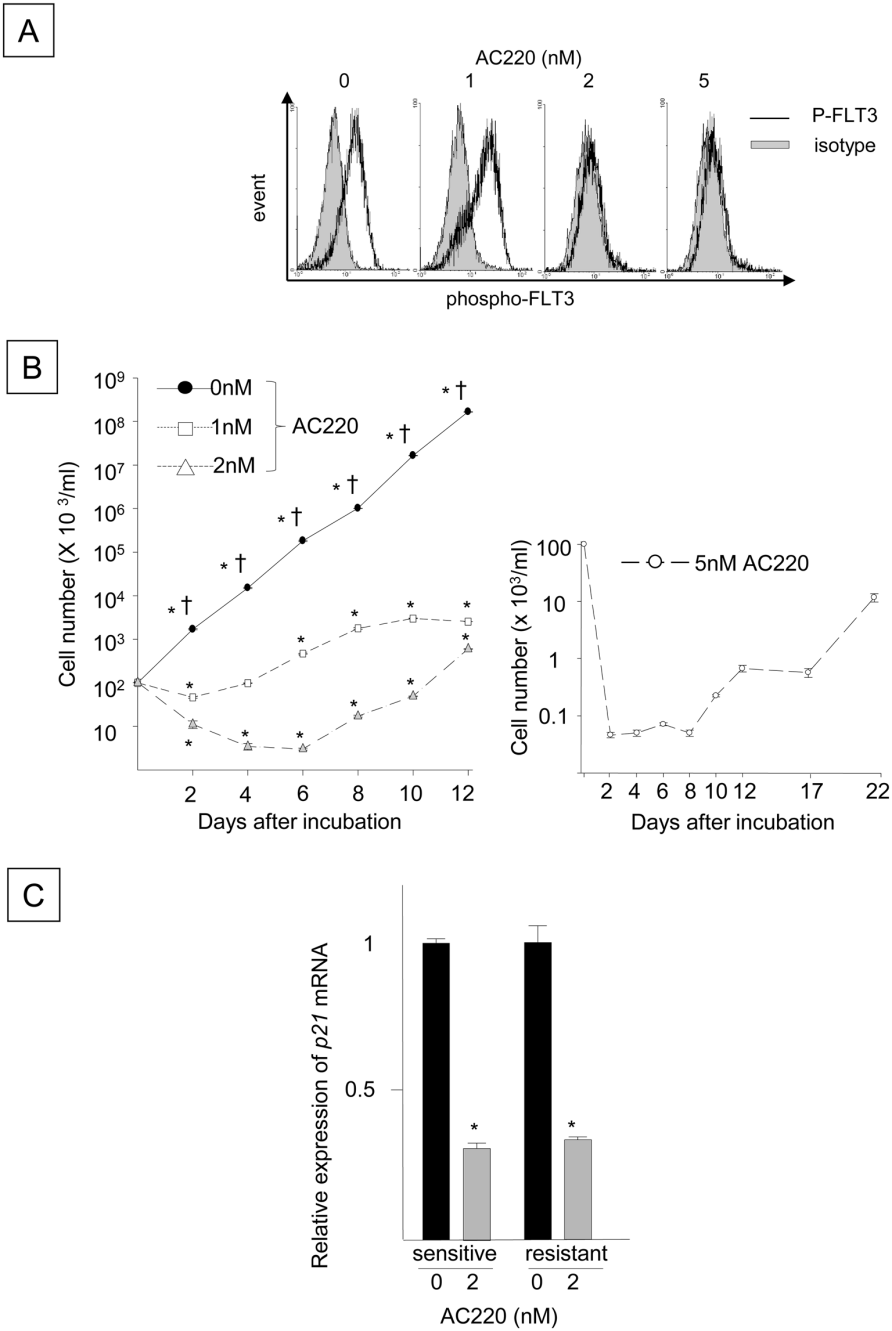
Blocking FLT3-ITD Using AC220 Decreases p21 Expression Coincident with Emergence of FLT3-ITD^+^ Cells Refractory to AC220. (A) The panel shows FLT3 phosphorylation in N51-FLT3-ITD-Ba/F3 cells incubated with 1, 2 or 5 nM AC220 for 24 hours. The cells were stained for phospho-FLT3 using a rabbit monoclonal antibody raised against Tyr591 of human FLT3, followed by staining with a PE-conjugated anti-rabbit IgG secondary antibody and flow cytometry analyses. (B) The left panel indicates the numbers of viable N51-FLT3-ITD-Ba/F3 cells incubated with 1 or 2 nM AC220 compared to the DMSO control (0 nM). Cells plated at a density of 1 x 10^5^ cells/ml were incubated with 1 or 2 nM AC220 or control DMSO for 12 days. The right panel represents the number of viable cells that were incubated with 5 nM AC220 for 22 days. The medium was replaced every 5 days and contained 5 nM fresh AC220. The cells were enumerated using the trypan blue exclusion assay. The y-axis represents the cell number on the log scale. The data shown represent one of the three experiments that were analyzed in triplicate with identical results (*: P < 0.05 compared to time 0, †: P < 0.05 compared to 1 or 2 nM AC220). (C) The relative expression of p21 in N51-FLT3-ITD-Ba/F3 cells that were sensitive or refractory to AC220 and incubated with 2 nM AC220 compared to those incubated with the DMSO control. The refractory cells, which were established by incubation with 2 nM AC220, were washed and cultured for 48 hours in fresh medium without AC220. The cells were subsequently incubated with 2 nM AC220 for 48 hours. Similarly, parental cells that were sensitive to AC220 were incubated with 2 nM AC220 for 48 hours. The *p21* mRNA levels were quantitated before and after incubation with AC220 (*: P < 0.05, N = 3).

**Fig 5:**
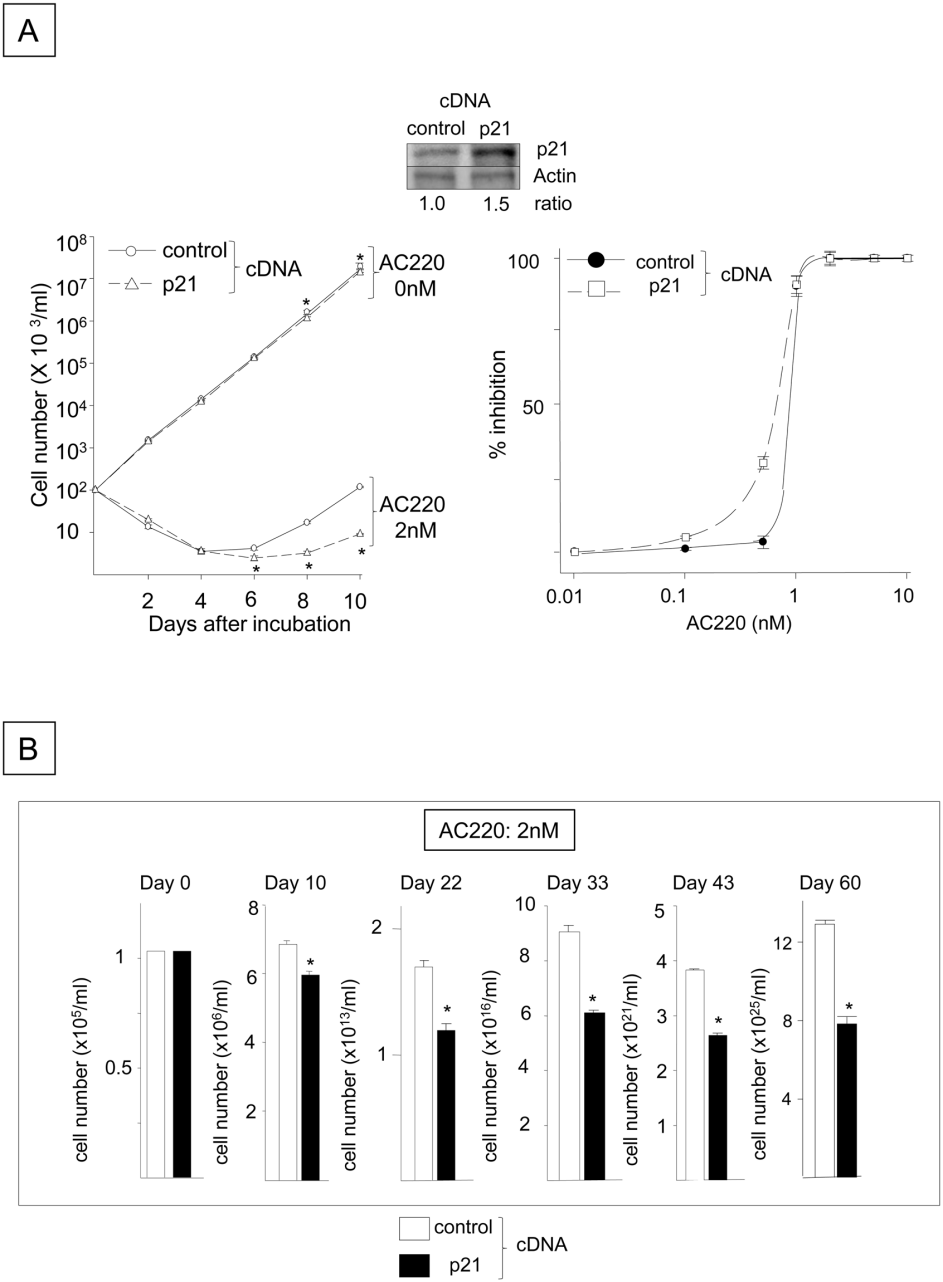
Overexpressing p21 Delays the Emergence and Proliferation of FLT3-ITD^+^ Cells Refractory to AC220. (A) The numbers of viable N51-FLT3-ITD-Ba/F3 cells transfected with the murine *p21* cDNA or control plasmid were quantitated in the presence or absence of 2 nM AC220. Cells were plated at a density of 1x10^5^ cells/ml and incubated with 2 nM AC220 or control DMSO for 10 days. The viable cells were enumerated using the trypan blue exclusion assay. The data shown represent one of three experiments that were analyzed in triplicate with identical results (*: P < 0.05 compared to the control plasmid). The expression levels of the p21 protein in the control cells and those transfected with the *p21* cDNA are shown above the histogram. The right panel indicates the AC220 dose-dependent inhibition of the control FLT3-ITD^+^ cells containing empty vector and those overexpressing p21. The cells were incubated with different concentrations of AC220 for 10 days, and the percent inhibition of viable cells was calculated compared to the control cells incubated with DMSO. (B) The panel indicates the numbers of viable FLT3-ITD^+^ cells containing the empty vector or p21 cDNA that were cultured in the presence of 2 nM AC220 for 60 days. The cells were plated at a density of 1x10^5^ cells/ml and were incubated with 2 nM AC220 or the DMSO control for 60 days. The medium was replaced every 5 days and contained 2 nM fresh AC220. The y-axes of the histograms are shown in linear but different scales (*: P < 0.05 compared to the control).

**Fig 6:**
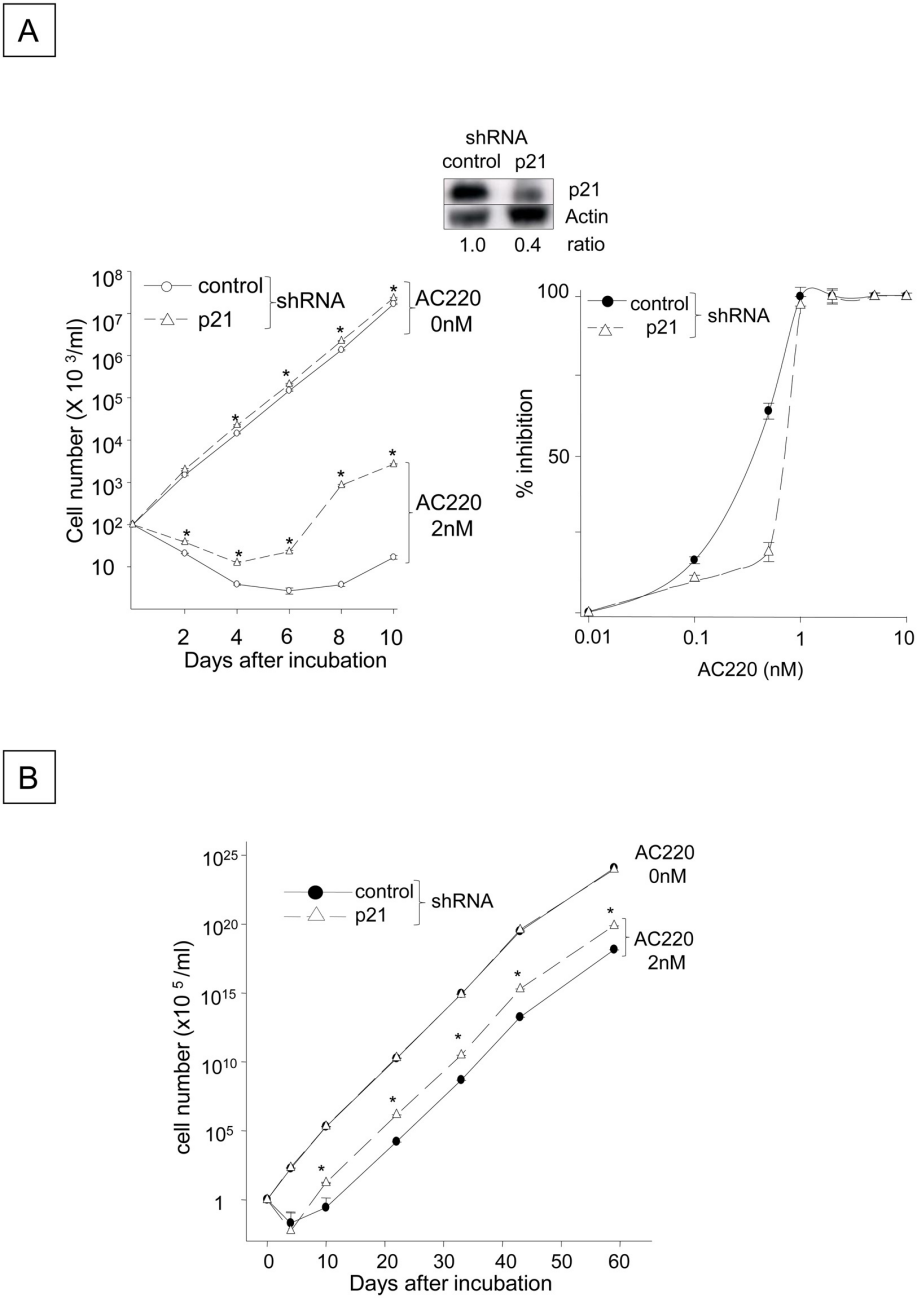
Silencing p21 Accelerates the Emergence and Proliferation of FLT3-ITD^+^ Cells Refractory to AC220. (A) The numbers of viable N51-FLT3-ITD-Ba/F3 cells transfected with the *p21* shRNA or control shRNA were quantitated in the presence or absence of 2 nM AC220. The cells were plated at a density of 1x10^5^ cells/ml and incubated with 2 nM AC220 or control DMSO for 10 days. The viable cells were enumerated using the trypan blue exclusion assay. The data shown represent one of three experiments that were analyzed in triplicate with identical results (*: P < 0.05 compared to the control shRNA). The expression levels of the p21 protein in both cell populations are shown in the inset (the same blot is shown in Fig 3D). The right panel indicates the AC220 dose-dependent inhibition of the control FLT3-ITD^+^ cells containing empty vector and those with shRNA for p21. The cells were incubated with different concentrations of AC220 for 10 days, and the percent inhibition of viable cells was calculated compared to the control cells incubated with DMSO. (B) The panel indicates the numbers of viable FLT3-ITD^+^ cells containing the empty vector or p21 shRNA that were cultured in the presence of 2 nM AC220 for 60 days. The cells were plated at a density of 1x10^5^ cells/ml and were incubated with 2 nM AC220 or the DMSO control for 60 days. The medium was replaced every 5 days and contained 2 nM fresh AC220.

The poor efficacy of FLT3-ITD inhibitors for FLT3-ITD^+^ AML [22,23] underscores the need to investigate the mechanism responsible for the drug-resistant phenotype of FLT3-ITD^+^ AML. The capacity of the FLT3-ITD-mediated up-regulation of p21 expression to inhibit the proliferation of FLT3-ITD^+^ HPCs suggests that FLT3-ITD signaling attenuates the proliferation and cell cycle progression of FLT3-ITD^+^ HPCs. However, the p21 down-regulation in FLT3-ITD^+^ cells triggered by inhibitors of FLT3-ITD, p53, PI3 kinase, and PKA suggests that the sustained inhibition of FLT3-ITD or other molecular pathways downstream of FLT3-ITD may block endogenous anti-growth signaling, possibly contributing to the recurrence of FLT3-ITD^+^ AML. To determine whether inhibiting p21 expression in FLT3-ITD^+^ cells by antagonizing FLT3-ITD is associated with the proliferation and/or emergence of FLT3-ITD^+^ cells that are refractory to a FLT3-ITD inhibitor, the association between p21 expression and cell proliferation was investigated in FLT3-ITD^+^ cells treated with a FLT3-ITD inhibitor. FLT3-ITD^+^ Ba/F3 cells were cultured in the absence of cytokines with 2 or 5 nM AC220, a selective inhibitor of FLT3-ITD, for 24 hours, and FLT3 phosphorylation was inhibited (Fig 4A). Although the reduced FLT3 phosphorylation was not evident after 24 hours of incubation with 1 nM AC220, prolonged incubation with 1, 2 or 5 nM AC220 significantly inhibited the growth-factor independent proliferation of FLT3-ITD^+^ Ba/F3 cells compared to the controls, suggesting that AC220 antagonizes the growth-stimulatory function of FLT3-ITD (Fig 4B). In contrast, the same treatment significantly decreased *p21* mRNA expression in the FLT3-ITD^+^ cells (Fig 4C), implying that FLT3-ITD inhibition disrupts growth-inhibitory signaling pathways, and not just pathways that stimulate cell proliferation. When the FLT3-ITD^+^ Ba/F3 cells were incubated with 1 or 2 nM AC220 for more than 8 days, cell proliferation began to increase despite the presence of AC220 (Fig 4B, left panel). Similarly, the number of viable cells declined immediately after incubation with 5 nM AC220, but cells began to proliferate after 21 days in the presence of 5 nM AC220 (Fig 4B, right panel), indicating the development of FLT3-ITD^+^ cells that were refractory to AC220, as previously reported [30]. Incubating the AC220-refractory cells with AC220 down-regulated p21 expression (Fig 4C), suggesting that AC220 can also disrupt growth-inhibitory signaling in AC220-refractory FLT3-ITD^+^ cells. However, p21 overexpression in the FLT3-ITD^+^ Ba/F3 cells significantly delayed the emergence of AC220-resistant FLT3-ITD^+^ cells (Fig 5A, left panel) and inhibited the proliferation of AC220-resistant FLT3-ITD^+^ cells cultured in the presence of 2 nM AC220 for up to 60 days (Fig 5B). The medium was replaced every 5 days, and fresh AC220 was added at a concentration of 2 nM; therefore, the emergence of resistant cells was not due to the loss of AC220 activity. The IC50 for the control FLT3-ITD^+^ cells was 0.77 nM, whereas that in the cells expressing ectopic p21 was 0.65 nM on day 10 (Fig 5A, right panel). In contrast, *p21* silencing with an shRNA significantly delayed the reduction in the number of viable FLT3-ITD^+^ Ba/F3 cells induced by AC220 (Fig 6A, left panel) and accelerated the development and proliferation of FLT3-ITD^+^ cells refractory to 2 nM AC220 up to 60 days (Fig 6B). The IC50 of AC220 for the FLT3-ITD^+^ cells containing the control shRNA was 0.35 nM on day 10, whereas p21 silencing increased the IC50 to 0.7 nM (Fig 6A, right panel). These findings suggest that the down-regulation of p21 resulting from FLT3-ITD inhibition facilitates proliferation and the development of FLT3-ITD^+^ cells that are refractory to AC220.

### The enhanced proliferation of FLT3-ITD-transformed HPCs following p21 deletion is associated with up-regulation of Pbx1 expression

**Fig 7:**
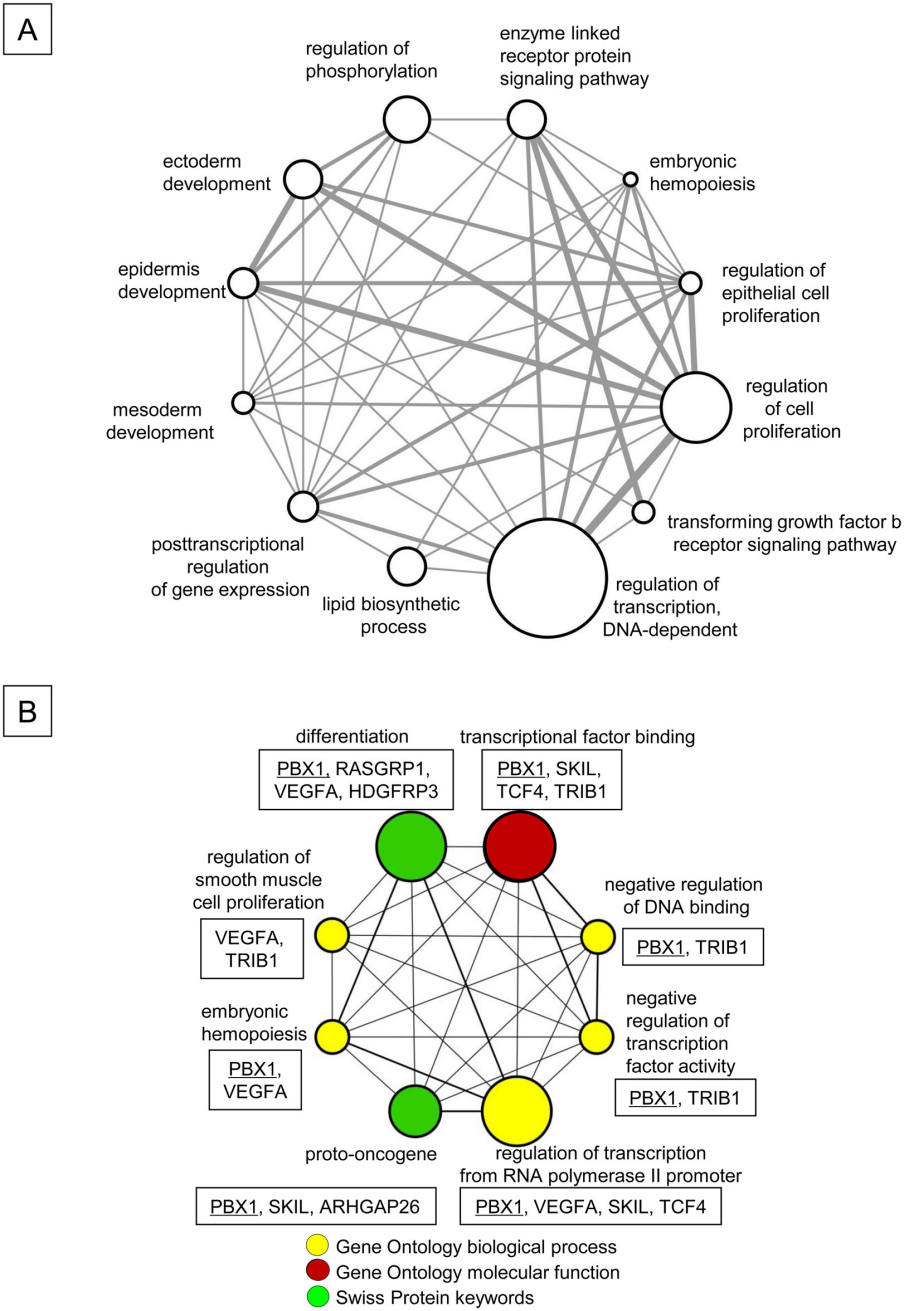
Enhanced Proliferation of FLT3-ITD^+^ KSL Cells by p21 Deletion is Associated with Alteration of Expression of Genes Linked to Various Biological Functions. (A) The functional classification of the 111 genes regulated by p21 in FLT3-ITD^+^ KSL cells. These 111 genes were classified based on the Biological Process subclass of the Gene Ontology database using the DAVID program [44]. The network of representative biological processes was visualized using Cytoscape [45]. The size of the circle indicates the number of genes in a given category, and the thickness of the bar represents the redundancy between the groups. All of the gene lists and functional categories are shown in S1 Table. The array data have been deposited in the NCBI Gene Expression Omnibus (GSE75200). (B) The functional classifications of the 12 genes that were regulated by p21 in FLT3-ITD^+^ KSL cells and deregulated in human AML-LSC [40]. The modulated genes were classified based on their biological processes and molecular functions, as defined by Gene Ontology terms and Swiss Protein keywords using the DAVID program [44]. Five of the 12 molecules were not classified. The 8 significantly enriched functional categories (P < 0.05) were connected and visualized using Cytoscape [45]. PBX1 was identified in 7 of 8 functional categories. The size of the circle indicates the number of genes in a given category, and the thickness of the bar represents the redundancy between the groups. The genes that were classified in each functional category are shown in the box. PBX1 is underlined. The fold changes in the expression levels of the listed genes are shown in Table 1.

In addition to regulating the cell cycle and apoptosis [1,2,6,7,13], p21 can modulate the activity of a number of transcription factors and co-activators [14–18], suggesting that it may regulate cell fate by influencing gene expression [19]. To identify the potential mechanisms by which p21 attenuates the proliferation of FLT3-ITD^+^ HPCs, we compared the gene expression profiles of p21^+/+^ and p21^-/-^ FLT3-ITD^+^ KSL cells. In three separate experiments, p21 deletion in FLT3-ITD^+^ KSL cells resulted in changes in the expression of 111 distinct mRNAs compared to the p21^+/+^ FLT3-ITD^+^ KSL cells, which were either unaffected or differentially regulated in normal bone marrow KSL cells (Table 1 and S1 Table). The 111 genes were functionally classified based on the biological process subclasses of the Gene Ontology database (Fig 7A). The top 3 functional groups that were associated with p21 include the regulation of transcription (DNA-dependent), cell proliferation and phosphorylation. Among the 111 genes, 12 were deregulated in human AML stem cells (AML-LSC) (Table 1) [40]. These 12 genes were further classified into 8 functional groups based on the Gene Ontology terms and the Swiss Protein database (Fig 7B). The transcription factor Pbx1, which is associated with HSC and HPC function [28,29] and is down-regulated in AML-LSC [40], was up-regulated in FLT3-ITD^+^ KSL cells upon p21 deletion. However, Pbx1 was not affected by p21 deletion in normal KSL cells (see below). Pbx1 itself was classified in 7 of the functional groups (Fig 7B).

**Table 1:** Fold Changes in the Genes Regulated by p21 Deletion in the FLT3-ITD-transformed KSL Cells.

Gene symbol	Name	Fold change after p21 deletion in FLT3-ITD^+^ KSL cells	Number of functional classification hits (as shown in Fig 7A)
Pbx1	Pre-B cell leukemia transcription factor 1	2.1	7
Vegfa	Vascular endothelial growth factor A	1.8	4
Trib1	Tribbles homolog 1 (*Drosophila*)	2.3	4
Skil	SKI-like	1.6	3
Tcf4	Transcription factor 4	2.1	2
Rasgrp1	RAS guanyl-releasing protein 1	-2.5	1
Hdgfrp3	Hepatoma-derived growth factor-related protein 3	1.7	1

P21 deletion in FLT3-ITD^+^ KSL cells resulted in the modulation of the expression levels of 111 mRNAs that were either unaffected or differentially regulated in normal bone marrow KSL cells. Of these 111 genes, 12 were deregulated in human AML-LSC [40]. These genes were classified based on Gene Ontology terms and the Swiss Protein database (as shown in Fig 7B). Pbx1 was classified into 7 of the functional groups. The fold changes in the expression levels of the genes listed in Fig 7B are shown in Table 1.

**Fig 8:**
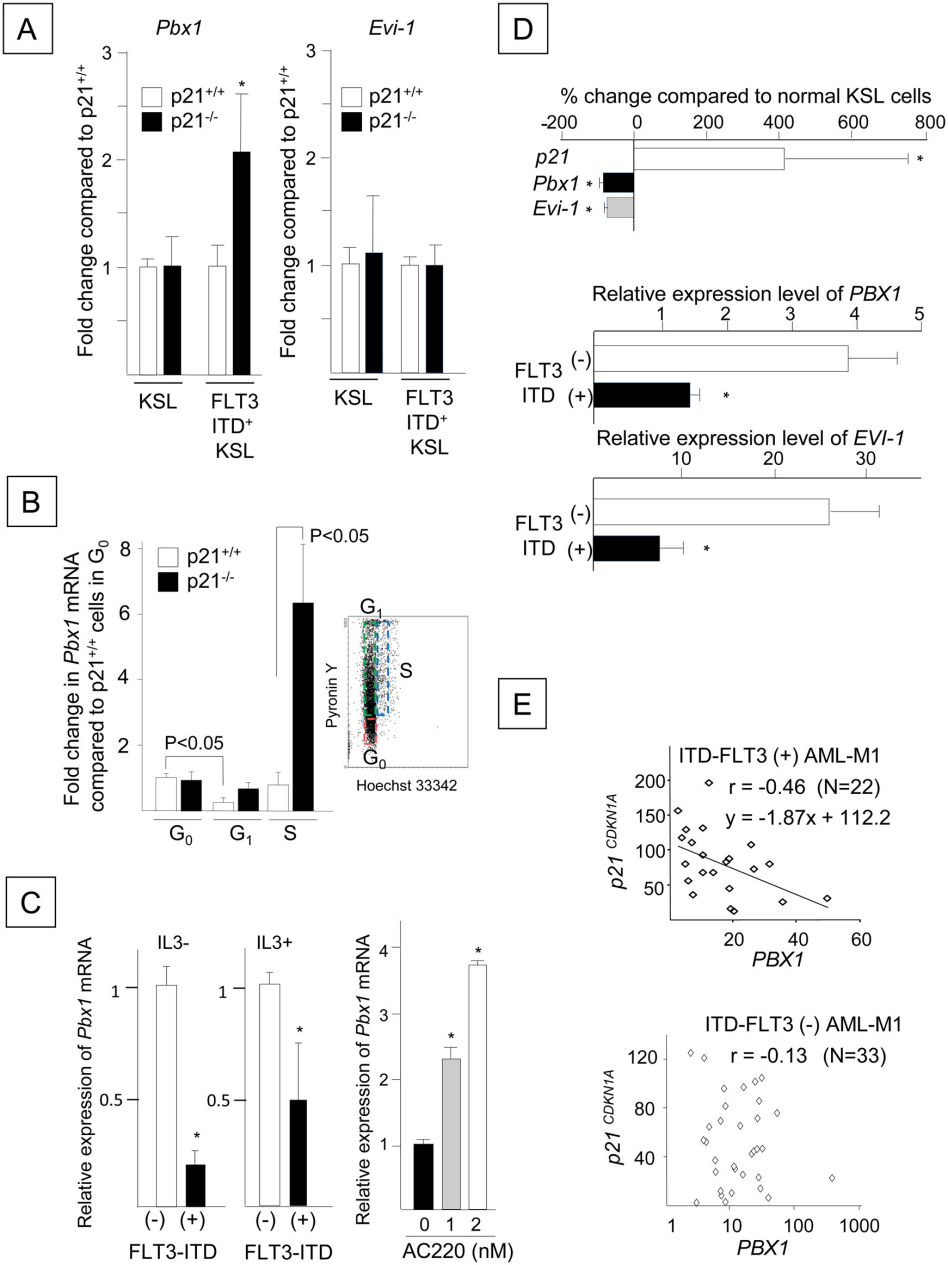
The Enhanced Proliferation of FLT3-ITD-transformed HPCs Following p21 Deletion is Associated with the Up-regulation of Pbx1 Expression. (A) The effects of p21 deletion on the *Pbx1* and *Evi-1* mRNAs. The left panel shows the expression levels of *Pbx1* mRNA in freshly isolated KSL cells or FLT3-ITD^+^ KSL cells, which were derived from p21^-/-^ cells, compared to p21^+/+^ cells. The expression levels of *Pbx1* relative to *Hprt* in p21^-/-^ KSL and p21^-/-^ FLT3-ITD^+^ KSL cells were compared to those in p21^+/+^ KSL and p21^+/+^ FLT3-ITD^+^ KSL cells, respectively, using quantitative RT-PCR (*: P < 0.001, N = 3). A similar analysis of *Evi-1* expression is shown in the right panel (NS, N = 3). (B) The expression levels of the *Pbx1* mRNA in p21^+/+^ and p21^-/-^ FLT3-ITD^+^ KSL cells at different stages of the cell cycle were compared. Marrow cells transduced with N51-*FLT3*-ITD were stained with antibodies for Sca-1, c-kit, and lineage markers, along with Hoechst 33342 and Pyronin Y. The relative expression levels of the *Pbx1* mRNA in FACS-sorted KSL cells in G_0_, G_1_ and S phases compared to p21^+/+^ cells in G_0_ phase are shown (N = 3). The sorting gate for cell cycle fractionation is shown in the inset. (C) The relative expression levels of *Pbx1* mRNA in Ba/F3 cells transduced with N51-*FLT3*-ITD compared to those transduced with wild-type *FLT3*. The left column shows *Pbx1* expression in cells without IL-3. The middle histogram presents the *Pbx1* mRNA levels in cells cultured with 0.1 ng/ml rmIL-3. The right histogram shows the *Pbx1* mRNA expression levels in N51-FLT3-ITD^+^ Ba/F3 cells incubated with 1 or 2 nM AC220 for 48 hours compared to the DMSO controls (*: P < 0.05, N = 3). (D) The upper panel shows the *p21*, *Pbx1* and *Evi-1* mRNA expression levels in FLT3-ITD^+^ p21^+/+^ KSL cells compared to normal KSL cells, as determined by quantitative RT-PCR. The percent changes in expression compared to the control KSL cells are shown (*: P < 0.01, N = 3). The middle and lower panels show the *PBX1* and *EVI-1* expression levels in human AML cells with or without FLT3-ITD. The *PBX1* (middle) and *EVI-1* (lower) expression levels in human FLT3-ITD^+^ AML cells (N = 78) were compared to those in the FLT3-ITD^-^ AML cells (N = 190) listed in GSE1159 (excluding patients with *FLT3*-TKD mutations, www.ncbi.nlm.nih.gov/geo/, *: P < 0.05). (E) The relationship between *p21* and *PBX1* in the AML M1 cases with (upper panel) or without (lower panel) FLT3-ITD that are listed in GSE1159 (www.ncbi.nlm.nih.gov/geo/) was analyzed using Microsoft Excel. The x-axes are displayed in linear and log scales in the top and bottom panels, respectively. Correlation values of r > 0.4 or < -0.4 were considered to indicate significant correlations between *p21* and *PBX1* (P < 0.05).

Although the loss of p21 did not significantly affect *Pbx1* expression in normal KSL cells (1.0 ± 0.3-fold change compared to p21^+/+^ KSL cells, NS), the quantitative RT-PCR analysis confirmed that *Pbx1* was up-regulated by 2.1 ± 0.5-fold in FLT3-ITD^+^ p21^-/-^ KSL cells compared to FLT3-ITD^+^ p21^+/+^ KSL cells (P < 0.001, Fig 8A, left panel). Because *Pbx1* expression is transcriptionally regulated by the transcription factor Evi-1 [41] and FLT3-ITD and because p21 altered Pbx1 expression, we hypothesized that the changes in *Pbx1* expression induced by FLT3-ITD and/or p21 are mediated by Evi-1. Therefore, we investigated the *Evi-1* levels in the freshly isolated KSL cells and the FLT3-ITD-transformed KSL cells in the presence or absence of p21. However, p21 deletion had no effect on *Evi-1* expression in normal or FLT3-ITD-transformed KSL cells (Fig 8A, right panel). The *Pbx1* mRNA levels in the p21^-/-^ KSL cells in G_0_ and G_1_ phases were equivalent to those in the p21^+/+^ cells (1.0 ± 0.3- and 1.4 ± 0.3-fold changes, respectively), whereas *Pbx1* expression in p21^-/-^ KSL cells in S phase was elevated by 6.1 ± 1.1-fold (P < 0.05, N = 3) compared to the p21^+/+^ cells (Fig 8B). Because *Pbx1* expression was not modulated by p21 in the absence of FLT3-ITD, we next determined the effect of FLT3-ITD on Pbx1 expression. FLT3-ITD^+^ Ba/F3 cells significantly down-regulated *Pbx1* mRNA levels compared to FLT3-ITD^-^ Ba/F3 cells (Fig 8C, left and middle panels). Conversely, the FLT3-ITD inhibitor AC220 significantly up-regulated *Pbx1* mRNA expression in the FLT3-ITD^+^ Ba/F3 cells (Fig 8C, right panel). Although the up-regulation of Pbx1 expression induced by the loss of p21 in FLT3-ITD^+^ KSL cells was independent of Evi-1 expression, the transduction of *FLT3*-ITD into primary mouse bone marrow resulted in significant decreases in the *Pbx1* and *Evi-1* mRNA levels compared to their levels in freshly isolated KSL cells (P < 0.01, N = 3, Fig 8D, upper panel). In addition, we found that the *Pbx1* and *Evi-1* expression levels were significantly reduced in the FLT3-ITD^+^ AML cells (N = 78) compared to FLT3-ITD^-^ AML cells (N = 190, P < 0.05, Fig 8D, middle and lower panels), which are listed in the GSE1159 dataset (www.ncbi.nlm.nih.gov/geo/) [42]. These results suggest that both p21 and FLT3-ITD down-regulate Pbx1 expression, although the effect of p21 on Pbx1 is dependent on FLT3-ITD. Consistent with the concomitant up-regulation of p21 expression and the down-regulation of Pbx1 expression, we found that *p21* expression (CDKN1A; 202284_s_at in GSE1159) was inversely correlated with *Pbx1* expression, but not with *Evi-1* expression, in FLT3-ITD^+^ AML subjects, particularly those with the FAB M1 subtype (r = -0.46, P < 0.01, 212151_at in GSE1159, Fig 8E, top panel), although this correlation was not evident in other AML subgroups. In contrast, the association between *p21* and *PBX1* was not observed in either FLT3-ITD^-^ AML cells (r = -0.13, GSE1159, Fig 8E, bottom panel) or normal human CD34^+^ cells (GSE2666, HG-U133A and GSE30029, www.ncbi.nlm.nih.gov/geo/) [43].

### Pbx1 knockdown abrogates the enhanced proliferation of FLT3-ITD^+^ HPCs induced by p21 deletion

**Fig 9:**
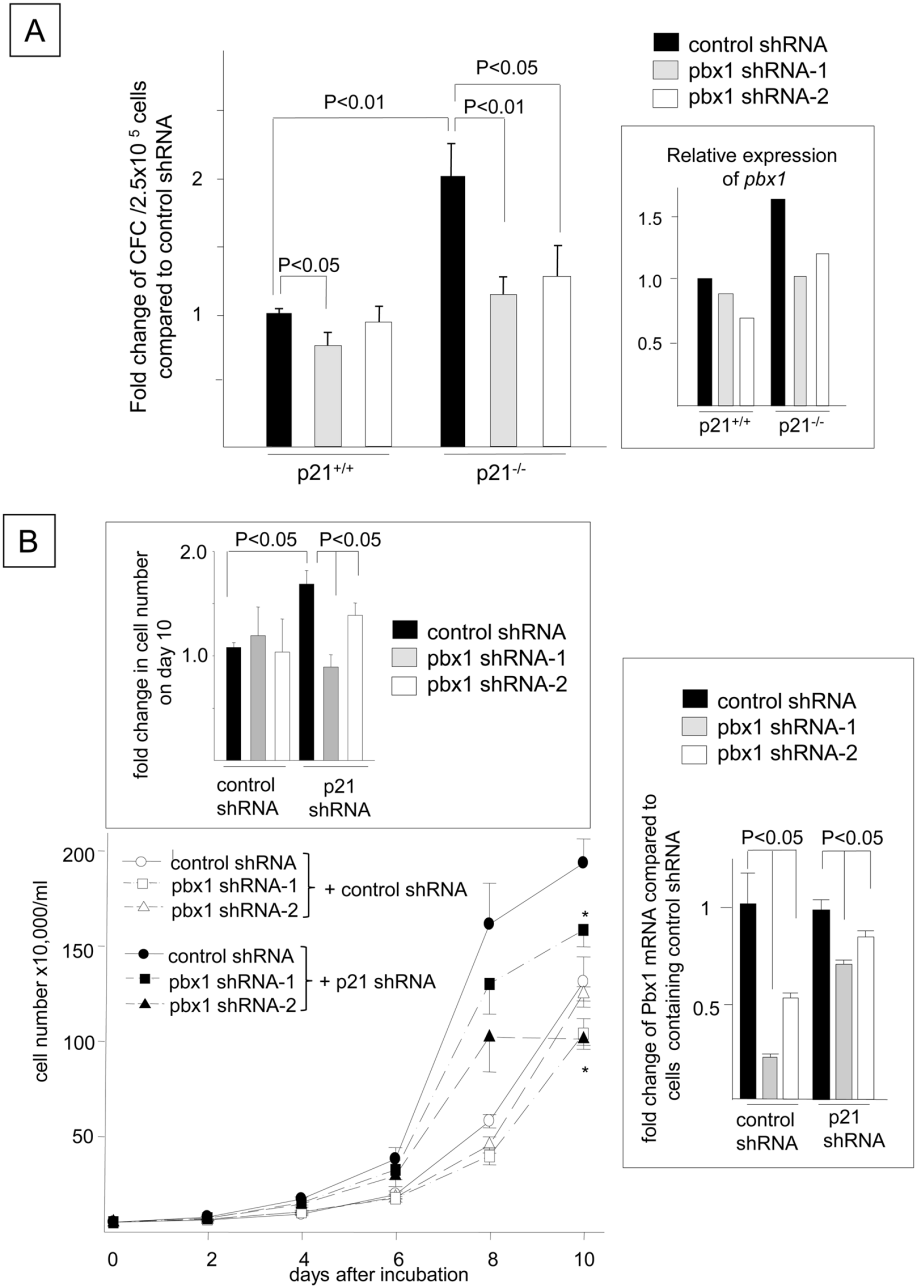
Pbx1 Knockdown Abrogates the Enhanced Proliferation of FLT3-ITD^+^ HPCs Induced by p21 Deletion. (A) Bone marrow cells from p21^+/+^ and p21^-/-^ mice were transduced with *FLT3*-ITD. Following the sorting of FLT3-ITD^+^ cells (using GFP as a marker) by FACS, the cells were transduced with a control shRNA or *Pbx1* shRNA (shRNA-1 or shRNA-2) using a pSINsi-mU6 plasmid, and the number of growth factor-independent CFCs grown in methylcellulose was quantified 14 days later. The data are shown as the fold changes in the number of CFCs compared to those of FLT3-ITD^+^ p21^+/+^ cells transduced with the control shRNA (N = 3). Identical results were obtained after 1 week of culture. Quantitative RT-PCR was performed to compare *Pbx1* expression relative to *Hprt* in the *Pbx1* shRNA-transduced samples and p21^+/+^ cells transduced with the control shRNA (inset). (B) FLT3-ITD^+^ Ba/F3 cells (N51) harboring a *p21* shRNA or control shRNA were transduced with a *Pbx1-*targeted shRNA or control shRNA. The cells were transfected with a p21 shRNA or a control shRNA and selected in the presence of 3 μg/ml puromycin for 2 weeks. The reduction in p21 expression was validated as shown in Fig 3D. The cells were then transfected with the control shRNA or two different shRNAs for Pbx1 (Pbx1 shRNA-1 and Pbx1 shRNA-2) and selected with 1 mg/ml G418 for 2 weeks. The reduction in *Pbx1* mRNA expression was validated by Q-RT-PCR and shown in the inset (right). The cells were cultured in 1% FBS/RPMI without any growth factors for 10 days and enumerated using the trypan blue exclusion assay. The data shown represent one of three experiments that were performed in quadruplicate with identical results (*: P < 0.01 compared to the control shRNA paired with the *Pbx1* shRNA). The top inset indicates the fold change in the cell number on day 10 compared to the FLT3-ITD^+^ Ba/F3 cells containing two control shRNAs. The inset on the right demonstrates the fold change in the *Pbx1* mRNA expression in the FLT3-ITD^+^ Ba/F3 cells transduced with the *Pbx1* shRNA compared to those transduced with the control shRNA paired with the *Pbx1* shRNA (N = 3, P < 0.05).

Because the enhanced proliferation of FLT3-ITD^+^ HPCs induced by the loss of p21 was accompanied by the up-regulation of Pbx1 expression, we next examined whether Pbx1 mediates the increased proliferation induced by p21 deletion. FLT3-ITD^+^ p21^-/-^ bone marrow cells transduced with a control shRNA showed a 1.9 ± 0.3-fold increase in the number of growth factor-independent CFCs compared to p21^+/+^ cells (P < 0.01, Fig 9A), which coincided with the up-regulation of *Pbx1* expression (Fig 9A, inset). The transduction of two different shRNAs targeting *Pbx1* into FLT3-ITD^+^ p21^-/-^ bone marrow cells resulted in reduced *Pbx1* mRNA levels (Fig 9A, inset) and CFC proliferation in the absence of hematopoietic growth factors compared to the control shRNA-transduced FLT3-ITD^+^ p21^-/-^ cells (Fig 9A, shRNA-1: 43 ± 4% reduction, P < 0.01, N = 3; shRNA-2: 41 ± 7% reduction, P < 0.05, N = 3). The number of CFCs resulting from the transduction of *Pbx1* shRNA into FLT3-ITD^+^ p21^-/-^ bone marrow cells was comparable to that generated by FLT3-ITD^+^ p21^+/+^ bone marrow cells that were transduced with the control shRNA (NS, N = 3). Similarly, transduction of the *Pbx1* shRNAs partially but significantly decreased the Pbx1 mRNA levels in the FLT3-ITD^+^ Ba/F3 cells containing the control shRNA, along with those harboring the p21 shRNA (Fig 9B, inset, right panel). The enhancement of the growth factor-independent proliferation of FLT3-ITD^+^ Ba/F3 cells induced by the *p21* shRNA was partially but significantly reduced by transducing these FLT3-ITD^+^ Ba/F3 cells with a *Pbx1* shRNA (N = 3, Fig 9B and the inset on the top). These data indicate that Pbx1 mediates the enhanced proliferation of growth factor-independent FLT3-ITD^+^ cells induced by p21 deletion.

## Discussion

**Fig 10:**
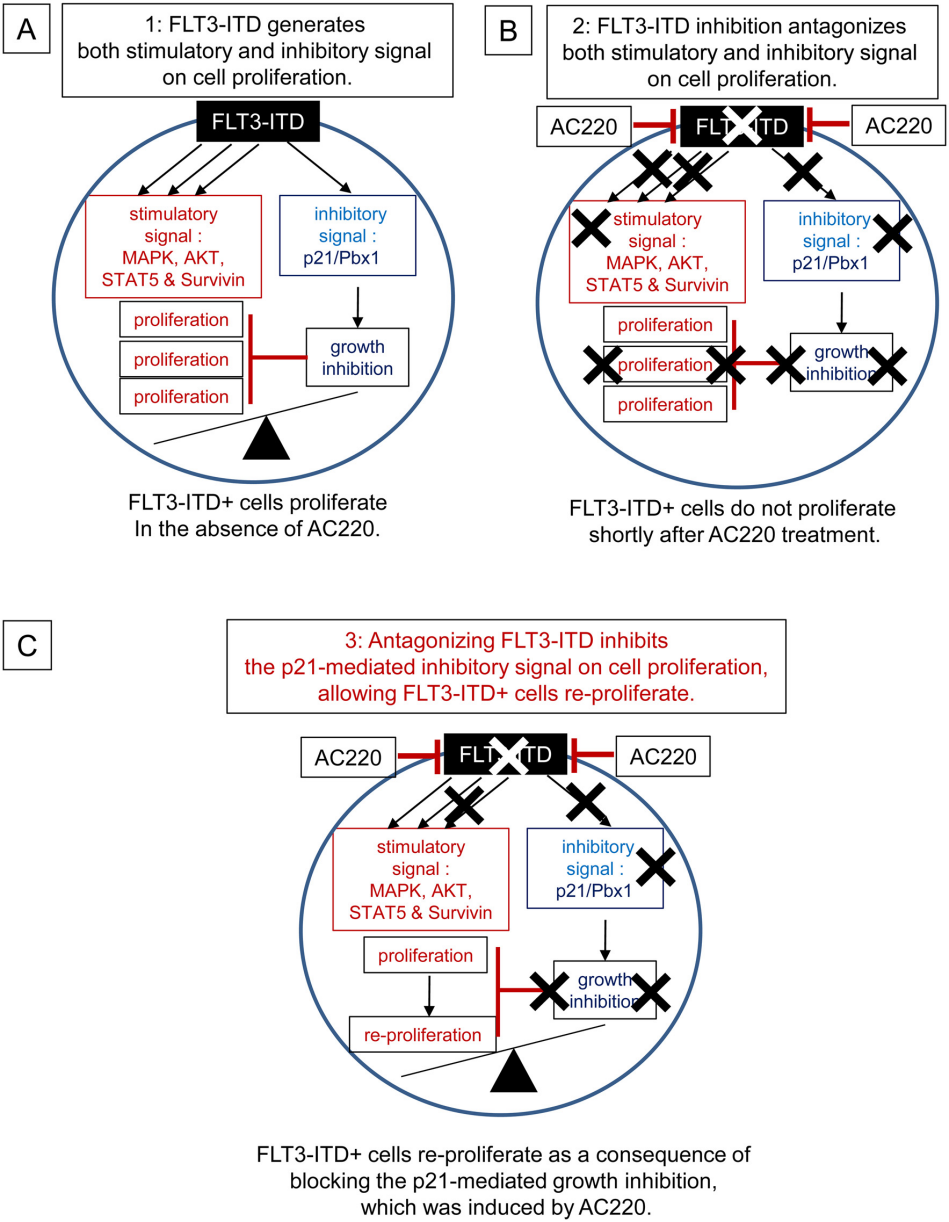
Suggested Roles of P21-mediated Inhibitory Signaling Pathways that Contribute to the Development of FLT3-ITD^+^ Cells that are Refractory to AC220. (A) FLT3-ITD stimulates various signaling molecules that promote cell proliferation or survival, such as MAP kinase, Akt, Stat5 and Survivin [20, 21, 30]. The present study shows that FLT3-ITD also generates a growth-inhibitory signal through p21, which is partially mediated by Pbx1. Because the signaling activity that stimulates cell proliferation or survival dominates the p21-mediated inhibitory signal, the FLT3-ITD^+^ cells proliferate in the absence of a FLT3-ITD antagonist. (B) Antagonizing FLT3-ITD with AC220 can not only eradicate the growth-stimulatory signals but also disrupt the growth-inhibitory signals by inhibiting p21 expression. FLT3-ITD^+^ cells do not proliferate immediately after incubation with AC220. (C) Prolonged incubation of FLT3-ITD^+^ cells with AC220 allowed the cells to re-proliferate, indicating that the cells acquire resistance to AC220, which is most likely mediated by the activation of stimulatory signals and/or disrupting the growth-inhibitory signals. Our data show that antagonizing FLT3-ITD with AC220 can decrease p21 expression and functions as a growth-inhibitory signal. Although p21 inactivation is not sufficient to initiate proliferation and other pro-survival pathways are required, the lack of p21 facilitates the development and proliferation of AC220-refractory cells. The mechanism responsible for the activation of the pro-survival pathways includes additional mutations of the FLT3-ITD gene, microenvironment-mediated resistance, or autocrine or paracrine stimulation of FLT3-ITD by the FLT3-ligand [22, 23]. These data strongly suggest that the disruption of p21 expression by FLT3-ITD inhibition facilitates the development and proliferation of AC220-resistant FLT3-ITD^+^ cells. Treatments targeting FLT3-ITD can potentially contribute to the refractory phenotype of FLT3-ITD^+^ AML cells toward FLT3-ITD inhibitors by eradicating the growth-inhibitory signals.

The present study shows that *FLT3*-ITD mutations produce proliferative and anti-growth signals (Fig 10A). FLT3-ITD concomitantly up-regulated p21 expression and down-regulated Pbx1 expression. The loss of p21 enhanced the growth factor-independent proliferation and cell cycle progression of FLT3-ITD^+^ cells and their sensitivity to Ara-C, suggesting that the enhanced expression of p21 attenuates cell cycle progression and confers chemoresistance to FLT3-ITD^+^ cells. Furthermore, the p21 deficiency promoted the selective up-regulation of Pbx1 expression in FLT3-ITD^+^ KSL cells, whereas silencing Pbx1 expression partially abrogated the enhanced proliferation of FLT3-ITD^+^ HPCs induced by p21 deletion, indicating that the p21-mediated inhibition of the proliferation of FLT3-ITD^+^ HPCs is mediated, at least in part, by Pbx1. Importantly, the p21 down-regulation resulting from FLT3-ITD inhibition by AC220 accelerated proliferation and development of FLT3-ITD^+^ cells that were refractory to AC220, suggesting that treatments targeting FLT3-ITD can eradicate growth-inhibitory signals by inhibiting p21 expression, thereby potentially contributing to FLT3-ITD^+^ AML progression (Fig 10B & 10C).

Our results indicate that growth factor withdrawal reduces p21^Cdkn1a^ expression in Ba/F3-FLT3 cells while increasing p27 ^Cdkn1b^ expression, and FLT3 ligand and FLT3-ITD up-regulate p21 expression but down-regulate p27 ^Cdkn1b^ expression. These data clearly support the differential regulation of p21 and p27 expression in response to FLT3 signaling. The up-regulation of p21 expression by FLT3-ITD was mediated through the p53, PI3 kinase and PKA pathways. Our data showed that FLT3-ITD up-regulated p21 expression in HPCs and Ba/F3 cells, concomitant with their enhanced proliferation. However, the loss of p21 enhanced the proliferation of FLT3-ITD^+^ HPCs and Ba/F3 cells, whereas p21 overexpression resulted in the opposite effect, indicating that although FLT3-ITD enhances HPC proliferation, the concomitant increase in p21 expression attenuates their proliferation. Therefore, FLT3-ITD harbors both growth-inhibitory and growth-stimulatory activities (Fig 10A). FLT3-ITD AML cells are characterized by their high proliferation rate and leukocytosis in patients [20, 21]. The presence of growth-inhibitory signaling downstream of FLT3-ITD appears to be contradictory to the increased proliferation potential of FLT3-ITD^+^ AML cells. The majority of the signaling components that exist downstream of FLT3-ITD are known to contribute to cell proliferation [20,21,30], suggesting that growth-stimulatory signaling dominates the p21-mediated inhibitory activity. The primitive KSL hematopoietic population exhibited the maximal increase in FLT3-ITD^+^ cell proliferation in response to the loss of p21. In contrast, the effect of the loss of p21 was minimal in differentiated cells, suggesting that p21 attenuates the proliferation of early undifferentiated cells, which resemble AML stem cells. This result is in good agreement with the increased cell cycle progression in HSCs, without altering the marrow cellularity or peripheral blood cell counts in p21-deficient mice [8]. A previous report showed that the induction of cell cycle entry eliminated AML stem cells in Ara-C-treated mice [39]. The enhanced cytotoxic effect of Ara-C in FLT3-ITD^+^ Ba/F3 cells induced by p21 silencing suggests that the up-regulation of p21 expression by FLT3-ITD may confer resistance to Ara-C by inducing quiescence in FLT3-ITD^+^ AML cells in the patients.

The down-regulation of p21 expression by AC220-mediated FLT3-ITD inhibition accelerated the development of FLT3-ITD^+^ cells that were refractory to AC220. AC220 down-regulated p21 expression in FLT3-ITD^+^ cells; however, p21 overexpression in FLT3-ITD^+^ cells significantly delayed the emergence of FLT3-ITD^+^ cells that were refractory to AC220, whereas p21 silencing resulted in the opposite effect. Our data suggest that the deregulation of p21 expression in FLT3-ITD^+^ cells contributes to chemoresistance in two distinct ways. FLT3-ITD^+^ cells undergo quiescence by up-regulating p21 expression, which enhances their resistance to conventional chemotherapeutic drugs, such as Ara-C. In contrast, the disruption of the expression and/or function of p21 resulting from FLT3-ITD inhibition contributes to the emergence of FLT3-ITD^+^ cells that are refractory to FLT3-ITD inhibitors. This result suggests that treatments that antagonize p21 function can sensitize FLT3-ITD^+^ cells to chemotherapy, whereas treatments that activate p21 may aid in inhibiting the development of FLT3-ITD^+^ cells that are refractory to FLT3-ITD inhibitors. Similar to FLT3-ITD-transformed cells, cells containing Bcr-Abl [46], AML1-ETO [4] and N-RasE12 [47], all of which regulate the proliferation of myeloid leukemia cells, exhibit increased p21 expression. The loss of p21 facilitates AML1-ETO-induced leukemogenesis [4] and increases the proliferation of Bcr-Abl-transformed hematopoietic cells [46], suggesting that these oncogenes also contain signaling components that negatively modulate the proliferation of leukemia cells. Moreover, the FLT3 ligand and stem cell factor provided by the bone marrow microenvironment [48] up-regulate p21 expression in Ba/F3-FLT3 cells and human AML cells [10, 11], respectively. Taken together, these data indicate that p21 expression that is up-regulated by the oncogenes and/or hematopoietic growth factors derived from the marrow microenvironment may contribute to the chemoresistance of AML cells.

Our data also indicate that the enhanced proliferation of FLT3-ITD^+^ cells induced by the loss of p21 was accompanied by the up-regulation of Pbx1 expression. Pbx1 is a product of a proto-oncogene that was originally discovered in acute leukemia and critically regulates organogenesis and hematopoiesis [28]. Pbx1 regulates the self-renewal of hematopoietic stem cells by maintaining their quiescence, whereas it facilitates the expansion of HPCs by regulating the function of common myeloid progenitor cells [28,29]. The hyperproliferation of FLT3-ITD^+^ HPCs and Ba/F3 cells induced by the concomitant loss of p21 and increased Pbx1 expression was partially abrogated by Pbx1 silencing, indicating that the enhanced proliferation of FLT3-ITD^+^ cells resulting from p21 deletion was due, at least in part, to the up-regulation of Pbx1 expression induced by p21 deletion. The up-regulation of Pbx1 expression induced by the p21 deletion implies that p21 down-regulates Pbx1 expression in FLT3-ITD^+^ cells. Interestingly, *Pbx1* silencing had little effect on the proliferation of FLT3-ITD^+^ cells containing the control shRNA. The data suggest that the involvement of Pbx1 in FLT3-ITD^+^ cells is likely more specific for the p21-mediated function, but not necessarily for the proliferation of FLT3-ITD^+^ cells in general. The finding that the p21 deletion had no effect on Pbx1 expression in normal KSL cells suggests that the modulation of Pbx1 expression by p21 is dependent on FLT3-ITD. Consistent with the FLT3-ITD-dependent modulation of Pbx1 expression following the loss of p21, FLT3-ITD decreased *Pbx1* mRNA expression in KSL and Ba/F3 cells, whereas AC220 up-regulated the *Pbx1* mRNA levels in FLT3-ITD^+^ Ba/F3 cells. Moreover, *PBX1* expression was significantly reduced in the FLT3-ITD^+^ AML cells compared to cells without *FLT3* mutations. Although FLT3-ITD down-regulated Evi-1, a transcriptional regulator of Pbx1 [41], p21 deletion had no effect on Evi-1 expression, irrespective of the FLT3-ITD status, suggesting that the p21-dependent modulation of Pbx1 expression is independent of Evi-1. Because p21 deletion in FLT3-ITD^+^ HPCs marginally increased the percentage of cells in S+G_2_/M phase and the expression of Pbx1 was higher in S phase than in G1 phase, Pbx1 up-regulation might be a consequence of cell cycle alterations induced by p21. A search of the chromosome 1 genomic contig of the *Mus musculus* strain C57BL/6J (MGSCv37 C57BL/6J: gi|149245775) uncovered a potential non-coding sequence in the 5’ region of the mouse *Pbx1* gene. A transcription factor-binding analysis using up to 2.0 kb of the identified 5’ non-coding sequence revealed candidate binding sequences for c-Myc, C/EBP-α and estrogen receptor-α, which are known to be directly regulated by p21 [15,17,18]. This result implies that c-Myc, C/EBP-α or estrogen receptor-α may be involved in the p21-induced transcriptional regulation of Pbx1.

## Conclusions

Our data demonstrate that FLT3-ITD attenuates FLT3-ITD^+^ cell proliferation by modulating the p21/Pbx1 axis. Although the FLT3-ITD-mediated increase in p21 expression increases resistance to Ara-C by reducing cell cycle progression, disruption of p21 expression using an FLT3-ITD inhibitor accelerates the development of refractory FLT3-ITD^+^ cells. This result suggests that the deregulated expression of p21 and/or Pbx1 by FLT3-ITD likely contributes to the refractory phenotype of FLT3-ITD^+^ AML cells. P21 and/or PBX1 may represent additional therapeutic targets for patients with FLT3-ITD^+^ AML, particularly those who are refractory to FLT3-ITD inhibitors.

## Supporting Information

S1 Table List of differentially regulated genes following p21 deletion in FLT3-ITD^+^ KSL cells compared to freshly isolated KSL cells. The genes that were exclusively up- and down-regulated by p21 deletion in the FLT3-ITD^+^ KSL cells but not the freshly isolated KSL cells are listed. The functional classifications of these genes based on Gene Ontology biological processes and molecular functions and KEGG pathways are also listed.
(XLSX)
